# Supplementary figures and images for: Evolutionary unique N-glycan-dependent protein quality control system plays pivotal roles in cellular fitness and extracellular vesicle transport in Cryptococcus neoformans
Source: eLife. 2025 May 27;13:RP103729. doi: 10.7554/eLife.103729 (PMC12113280; doi:10.7554/eLife.103729)

| Intracellular |       | Secretion |       | Intracellular |       | Secretion |       |
|---------------|-------|-----------|-------|---------------|-------|-----------|-------|
| WT            | ugg1Δ | WT        | ugg1Δ | WT            | ugg1Δ | WT        | ugg1Δ |

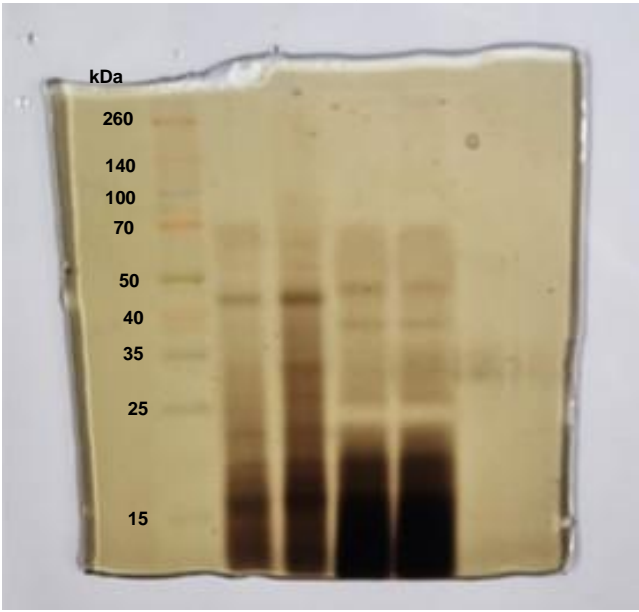

Supplement: Figure 2—source data 1. [file elife-103729-fig2-data1.pdf]

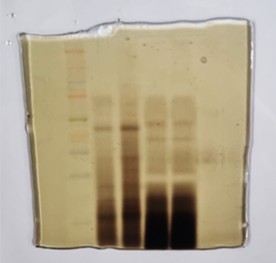

Supplement: Figure 2—source data 2. [file elife-103729-fig2-data2.jpg]

Intracellular      Secretion

\*

WT      *ugg1Δ*      WT      *ugg1Δ*

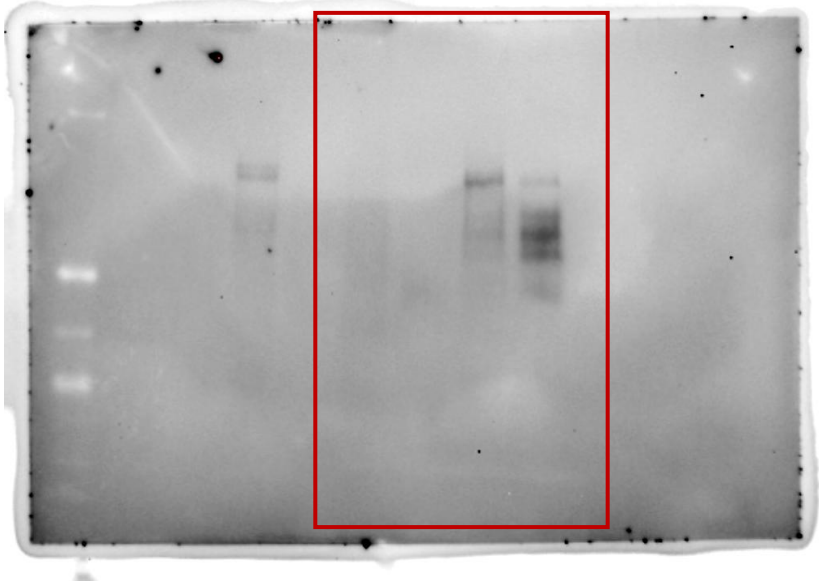

Supplement: Figure 2—source data 3. [file elife-103729-fig2-data3.pdf]

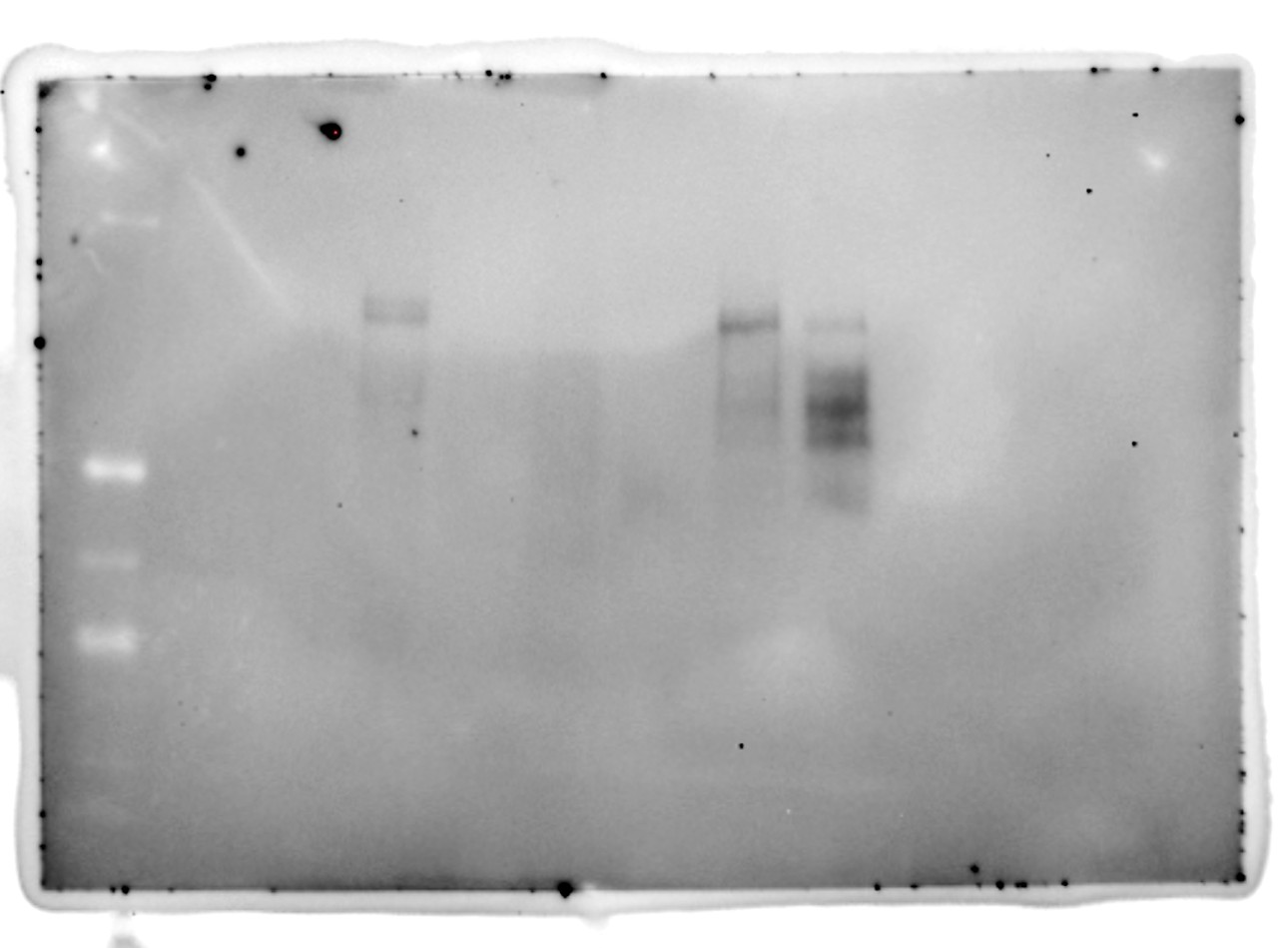

Supplement: Figure 2—source data 4. [file elife-103729-fig2-data4.jpg]

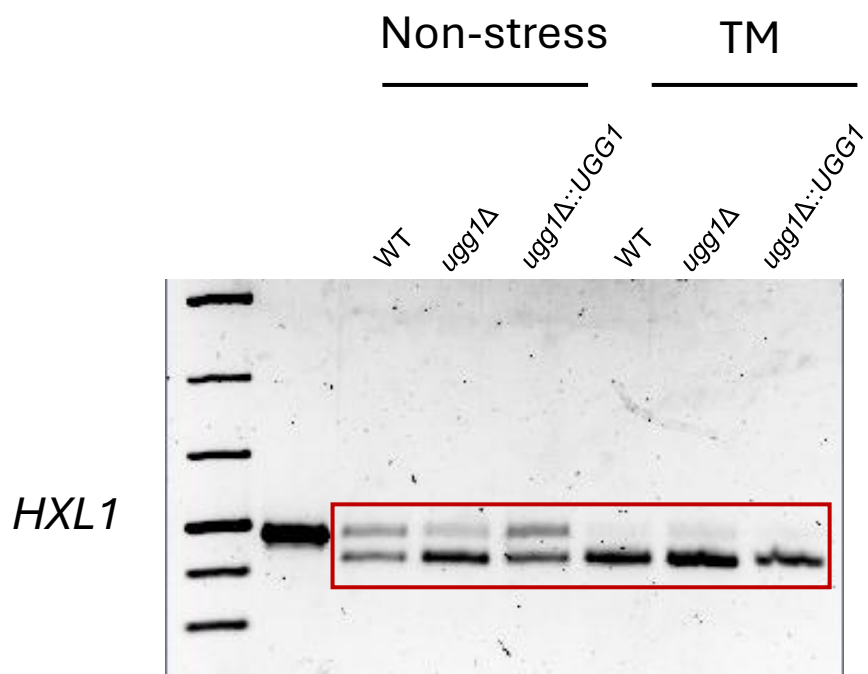

Supplement: Figure 3—source data 2. [file elife-103729-fig3-data2.pdf]

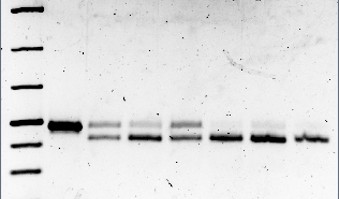

Supplement: Figure 3—source data 3. [file elife-103729-fig3-data3.jpg]

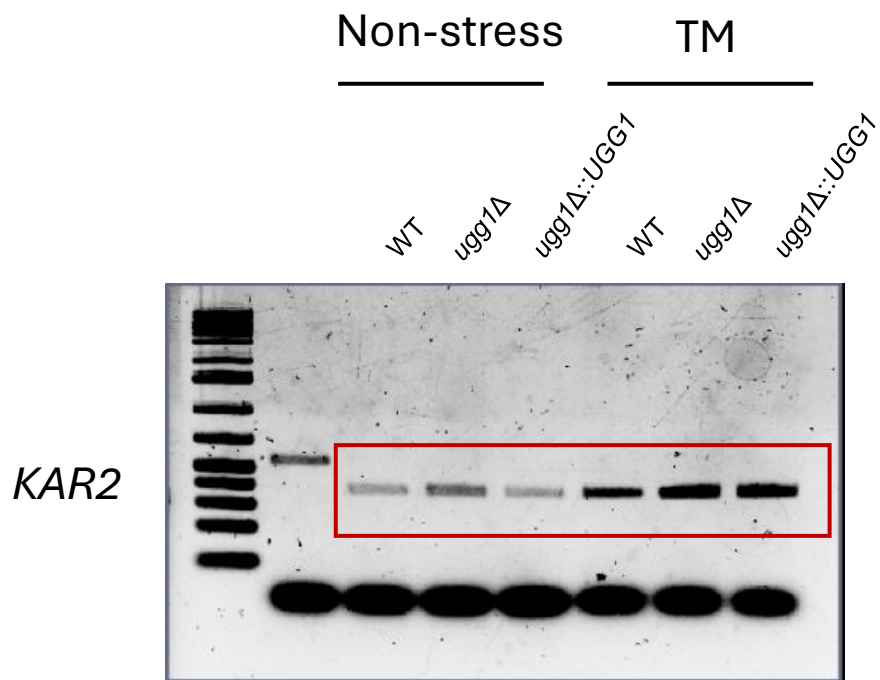

Supplement: Figure 3—source data 4. [file elife-103729-fig3-data4.pdf]

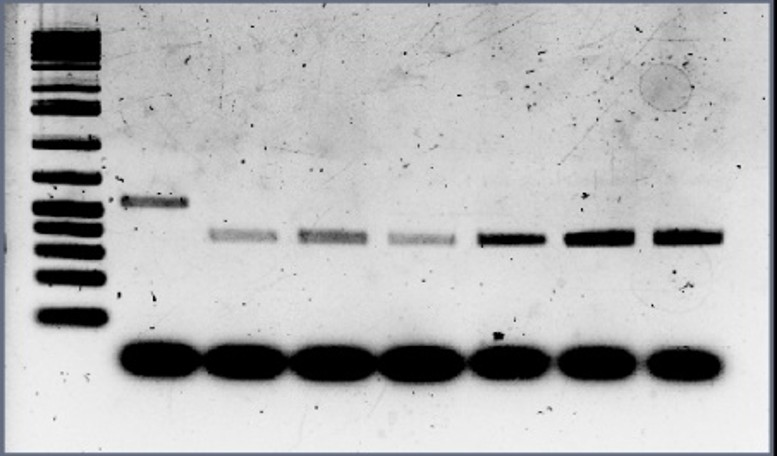

Supplement: Figure 3—source data 5. [file elife-103729-fig3-data5.jpg]

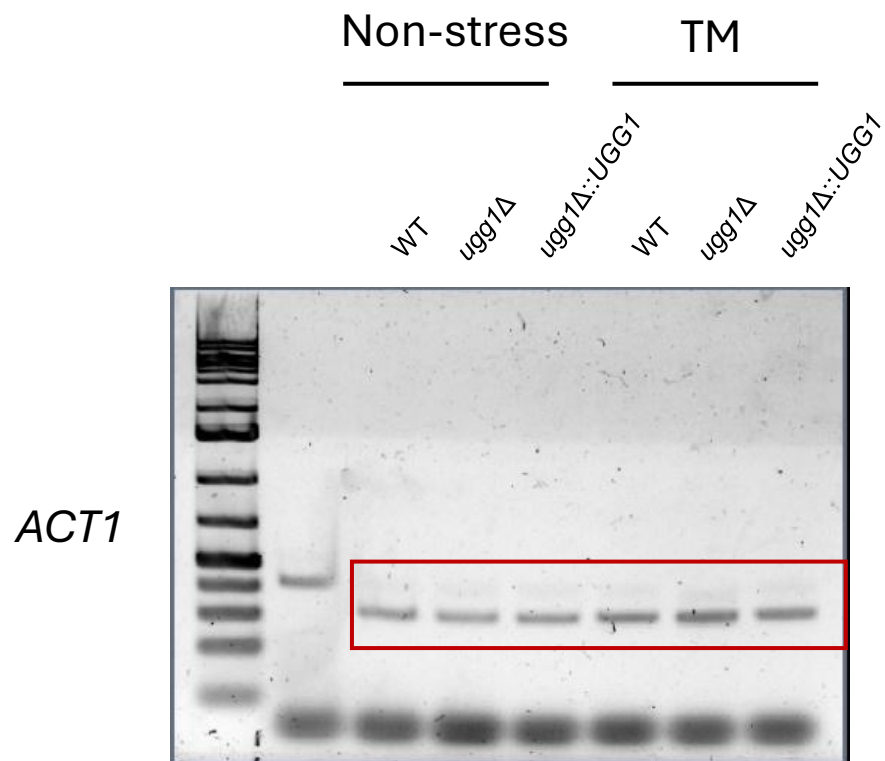

Supplement: Figure 3—source data 6. [file elife-103729-fig3-data6.pdf]

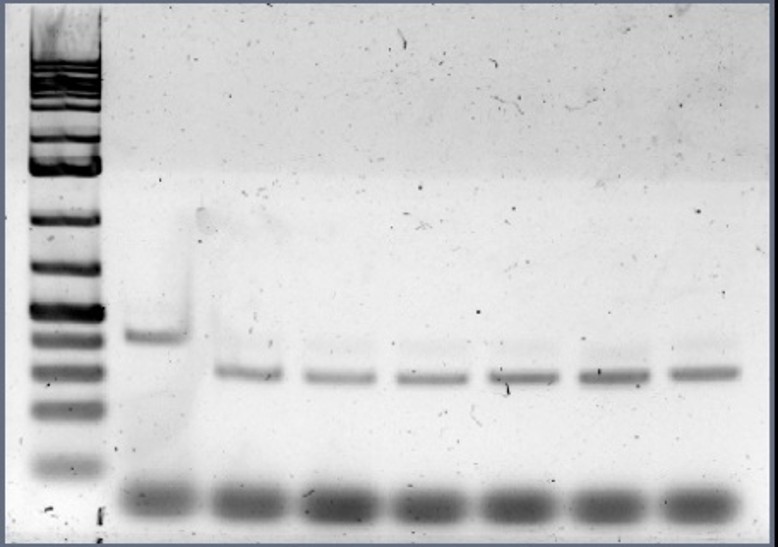

Supplement: Figure 3—source data 7. [file elife-103729-fig3-data7.jpg]

WT  
ugg1Δ  
mns1Δ101Δ  
ugg1Δ::UGG1  
rim101Δ  
cap59Δ

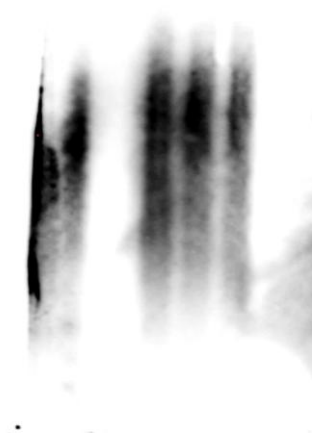

Intracellular GXM

Supplement: Figure 5—source data 2. [file elife-103729-fig5-data2.pdf]

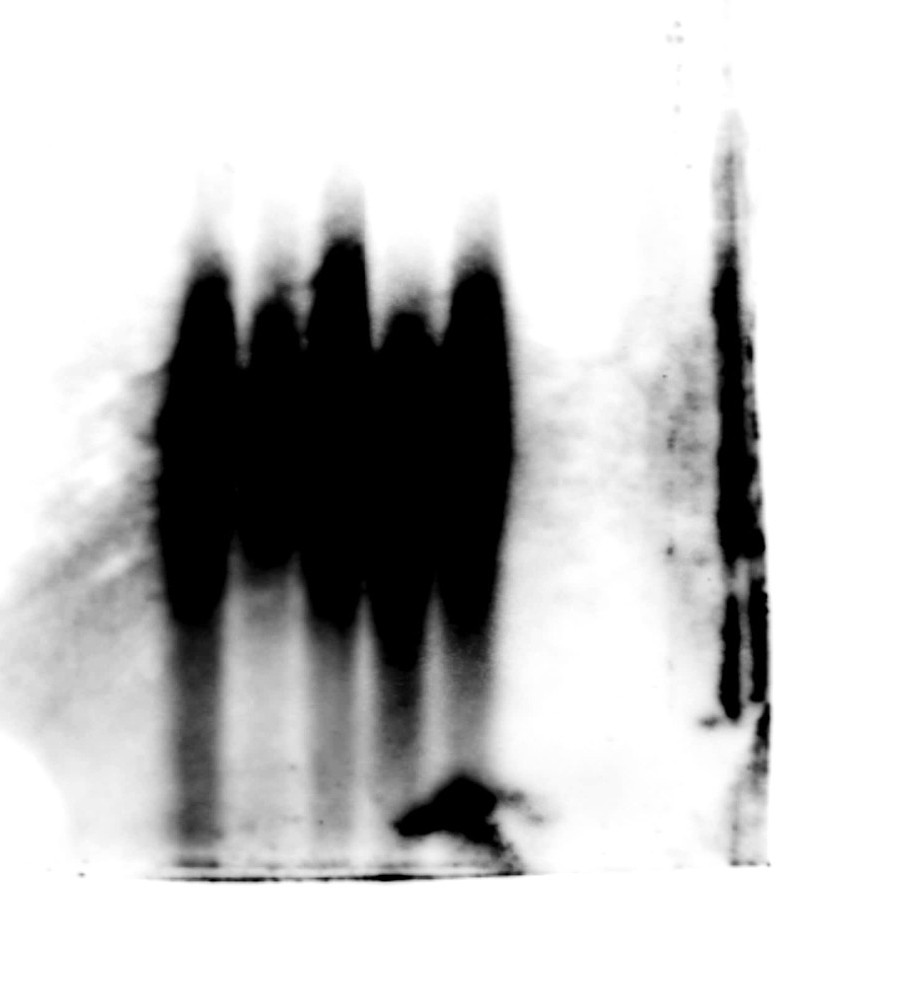

Supplement: Figure 5—source data 3. [file elife-103729-fig5-data3.jpg]

WT  
ugg1Δ  
mms1Δ101Δ  
ugg1Δ::UGG1  
rim101Δ  
cap59Δ

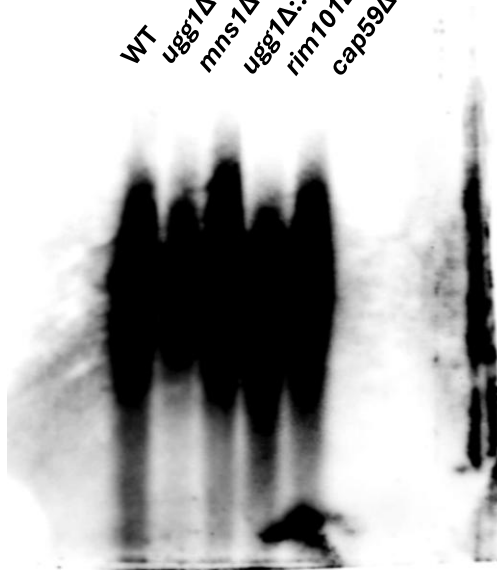

Shed GXM

Supplement: Figure 5—source data 4. [file elife-103729-fig5-data4.pdf]

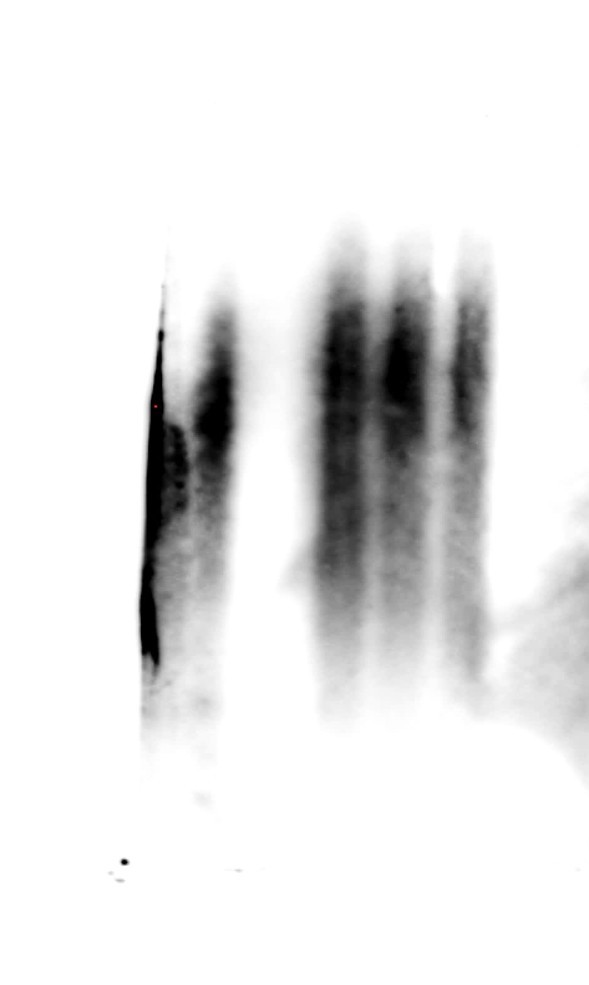

Supplement: Figure 5—source data 5. [file elife-103729-fig5-data5.jpg]

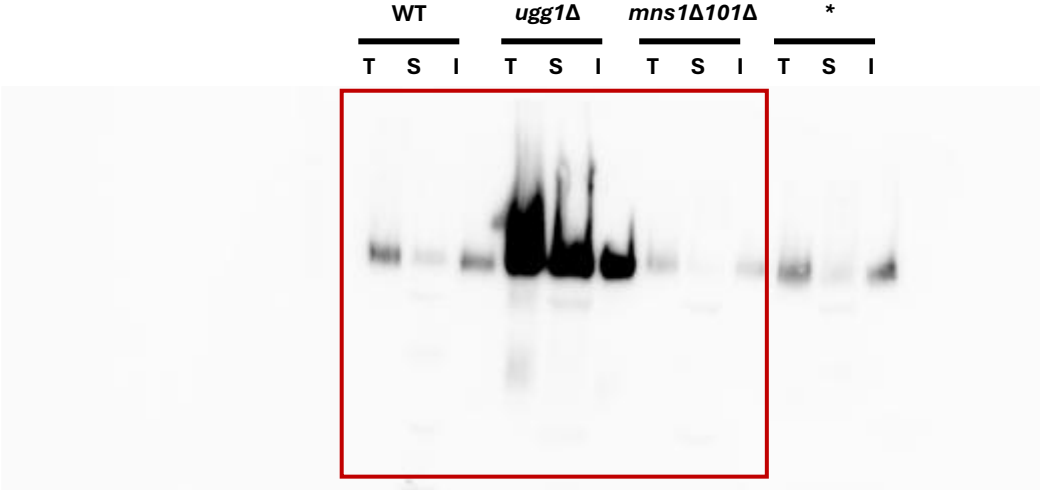

Intracellular

Supplement: Figure 7—source data 2. [file elife-103729-fig7-data2.pdf]

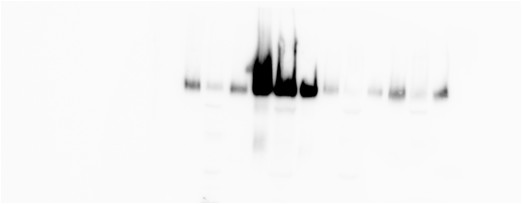

Supplement: Figure 7—source data 3. [file elife-103729-fig7-data3.jpg]

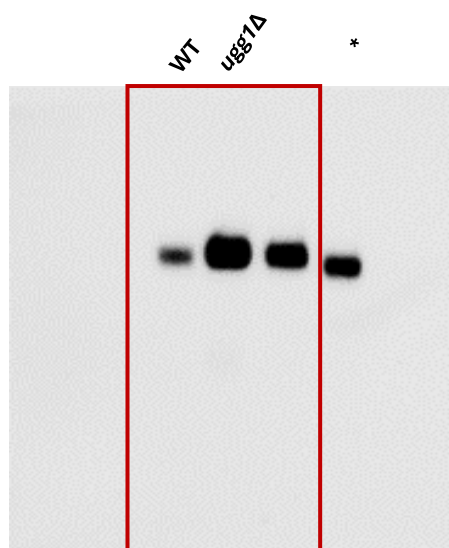

Secretion

Supplement: Figure 7—source data 4. [file elife-103729-fig7-data4.pdf]

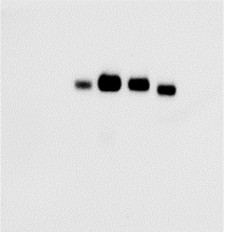

Supplement: Figure 7—source data 5. [file elife-103729-fig7-data5.jpg]

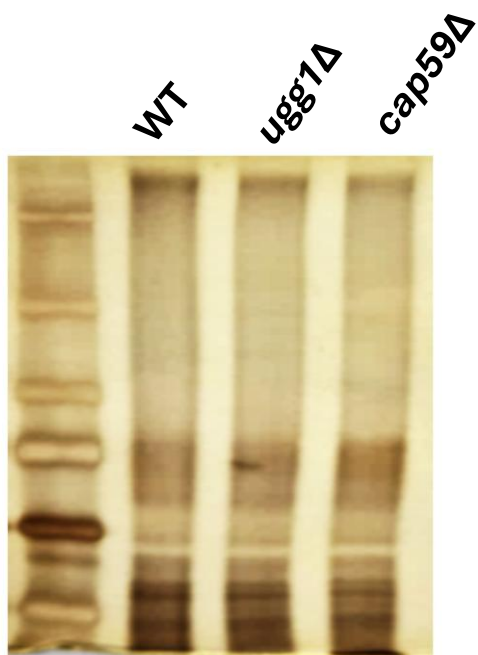

**Cell extract**

Supplement: Figure 8—source data 3. [file elife-103729-fig8-data3.pdf]

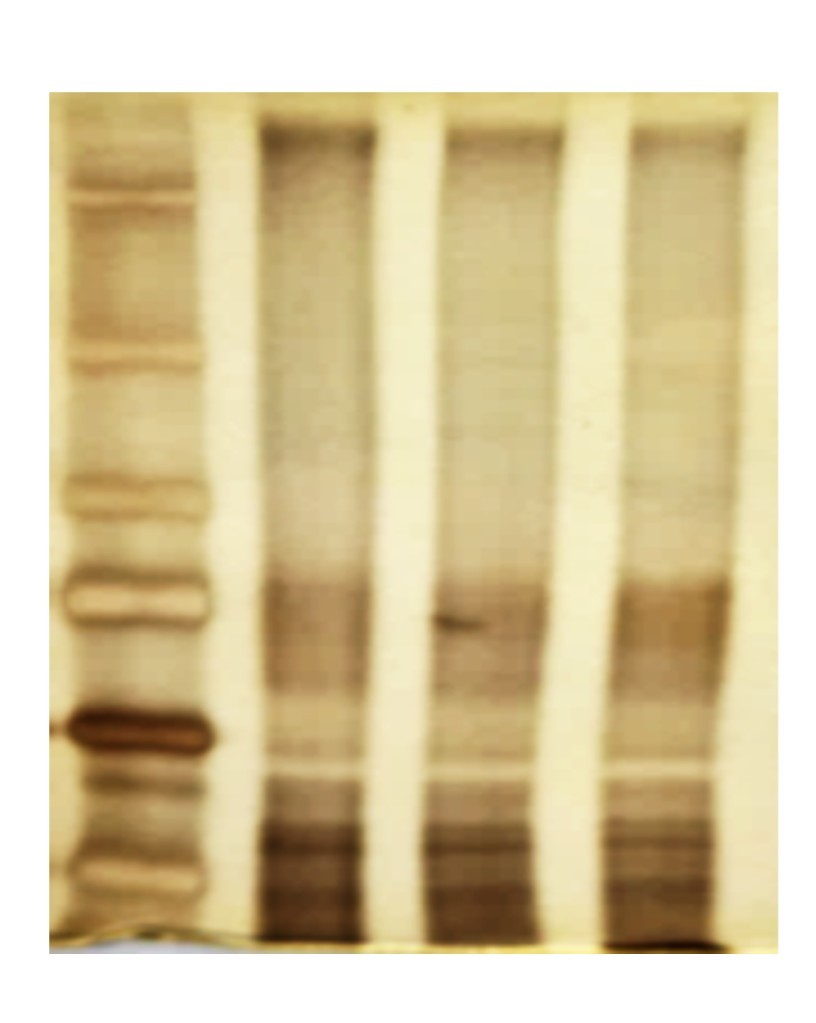

Supplement: Figure 8—source data 4. [file elife-103729-fig8-data4.jpg]

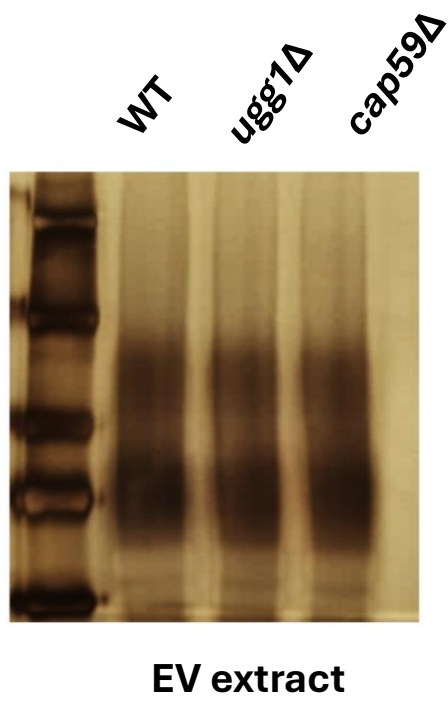

Supplement: Figure 8—source data 5. [file elife-103729-fig8-data5.pdf]

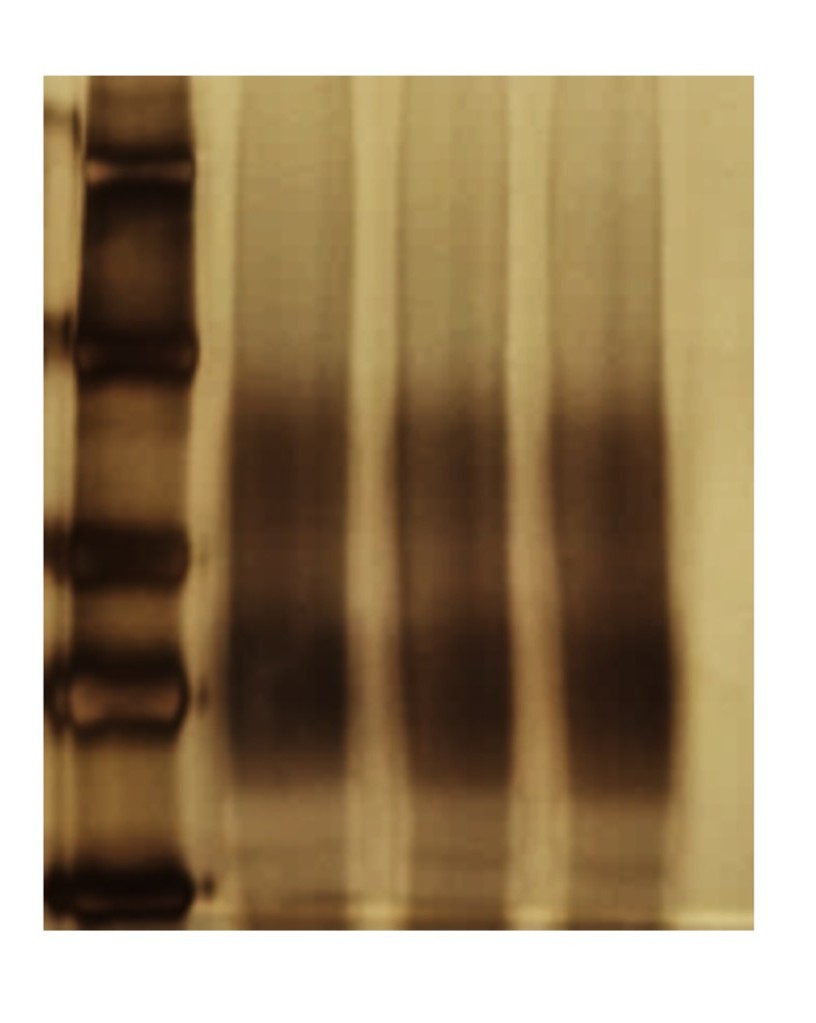

Supplement: Figure 8—source data 6. [file elife-103729-fig8-data6.jpg]

WT  
ugg1Δ  
cap59Δ

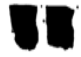

α- GXM

Supplement: Figure 8—source data 7. [file elife-103729-fig8-data7.pdf]

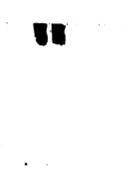

Supplement: Figure 8—source data 8. [file elife-103729-fig8-data8.jpg]

WT *ugg1Δ* *cap59Δ*

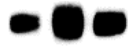

$\alpha$ - Cda1

Supplement: Figure 8—source data 9. [file elife-103729-fig8-data9.pdf]

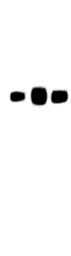

Supplement: Figure 8—source data 10. [file elife-103729-fig8-data10.jpg]

WT  
ugg1Δ  
cap59Δ

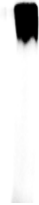

α- GXM

Supplement: Figure 8—source data 11. [file elife-103729-fig8-data11.pdf]

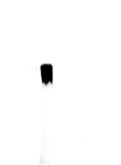

Supplement: Figure 8—source data 12. [file elife-103729-fig8-data12.jpg]

WT *ugg1Δ* *cap59Δ*

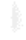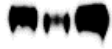

$\alpha$ - Cda1

Supplement: Figure 8—source data 13. [file elife-103729-fig8-data13.pdf]

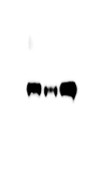

Supplement: Figure 8—source data 14. [file elife-103729-fig8-data14.jpg]
